# How to Distinguish Nonexponentiality and Nonlinearity in Isothermal Structural Relaxation of Glass-Forming Materials

**DOI:** 10.1021/acs.jpcb.4c02226

**Published:** 2024-08-08

**Authors:** Jiří Málek

**Affiliations:** Department of Physical Chemistry, Faculty of Chemical Technology, University of Pardubice, Studentská 573, Pardubice 532 10, Czech Republic

## Abstract

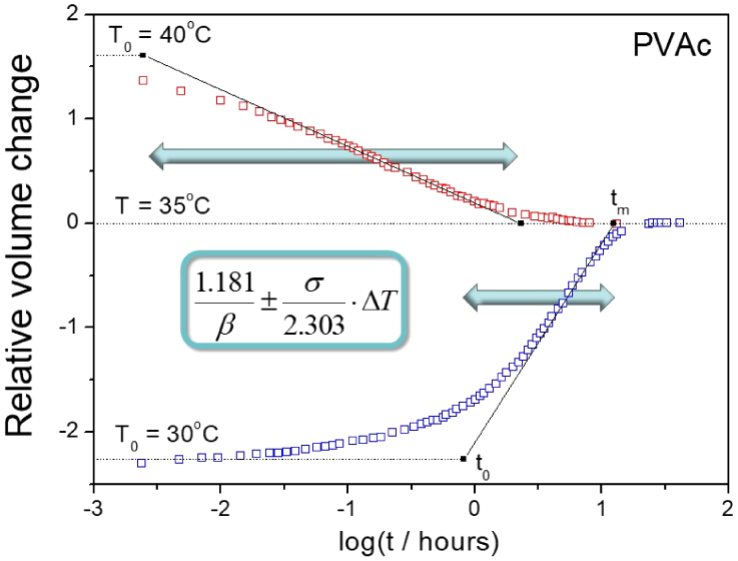

The nonexponentiality and nonlinearity are two essential features of the structural relaxation in any glass-forming material, which seem to be inextricably bound together by the material time. It is shown that the temperature down-jump and up-jump experiments of the same magnitude Δ*T* = *T*_0_ – *T* to the same temperature *T* provide a clue for their separation. The isothermal structural relaxation can be quantified using the stabilization period on the logarithmic time scale log(*t*_m_/*t*_0_). It is described as the sum of the nonexponentiality term 1.181/ß and the nonlinearity term (σ/2.303)Δ*T* for the temperature down-jump, and as their difference for the temperature up-jump. The material parameter σ = −(∂lnτ/∂*T*_f_)_i_ quantifies variation of the relaxation time with structural changes at the inflection point of the relaxation curve and is formulated for the most widely used phenomenological models. The asymmetry of approach to equilibrium after the temperature down-jump and up-jump was first described by Kovacs in 1963. A detailed analysis of this asymmetry is provided, and a simple method for the estimation of the parameters characterizing the nonexponentiality (ß) and nonlinearity (σ) is proposed. The applicability of this method is tested using previously reported isothermal experimental data as well as calculated data for aging of polymers and other glass-forming materials. This concept illuminates differences in structural relaxation kinetics in a simple and consistent way that can be useful in the design of novel materials and the evaluation of their physical aging treatment.

## 1 Introduction

**Figure 1:**
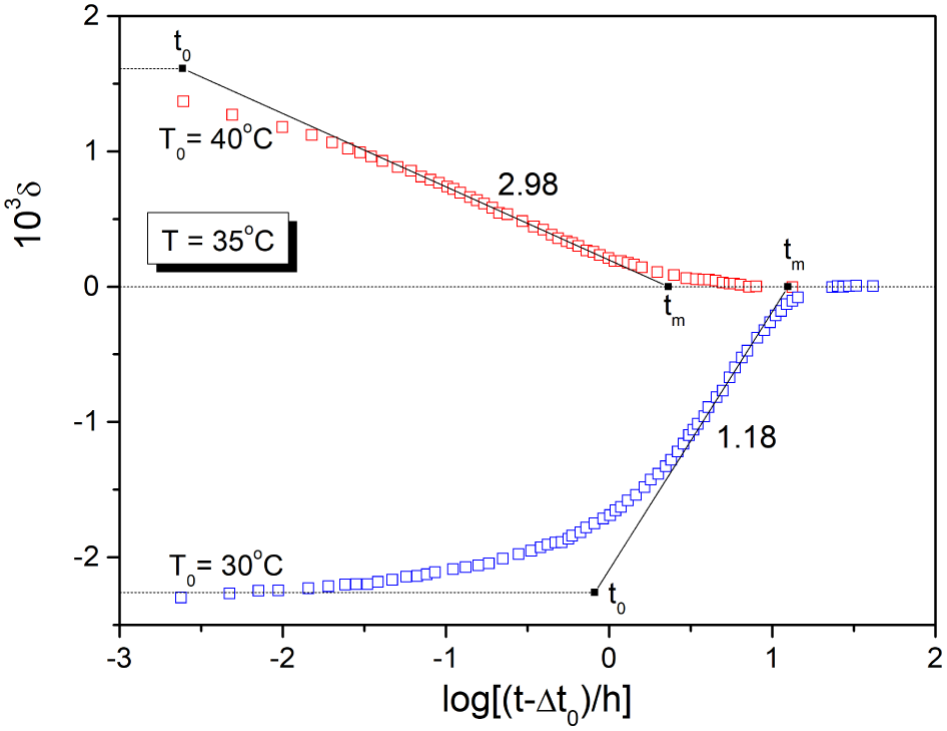
Asymmetry of the approach to equilibrium for the PVAc polymer, subjected to a temperature down-jump and up-jump of the same magnitude |Δ*T*| = 5 K. Data (points) and inflectional tangents (dδ/d log*t*)_i_ (solid lines) were taken from ref. 1, Δt_0_ = 36 s is the thermal equilibration time. The dotted lines indicate the initial departure δ_0_ at *T*_0_ = 40 and 30 °C as well as the equilibrium line at 35 °C. The numbers shown next to the curves correspond to the stabilization period calculated by eq 1.

Sixty years ago, Kovacs^1^ reported a well-known dilatometric experiment clearly demonstrating asymmetry of approach to equilibrium of amorphous polyvinyl acetate (PVAc). Isothermally monitored relative volume change δ = (V – V_∞_)/V_∞_ after a temperature down-jump and temperature up-jump of the same magnitude Δ*T* = *T*_*0*_ – *T* has very different dependences on a logarithmic time scale as they approach metastable equilibrium, as shown by open points in Figure 1. These experiments provide valuable information about out-of-equilibrium dynamics. The δ (log*t*) data exhibits clearly asymmetric response, resulting in a more stretched isothermal aging curve after temperature down-jump than after the temperature up-jump of the same size. This is a hallmark of physical aging (*Kovacs signature*) that has been discussed in recent perspective papers.^2,3^

The maximum rate of relative volume change for PVAc is observed at the inflection point, as indicated by the lines in Figure 1, originally drawn by Kovacs.^1^ These inflectional tangents (dδ/dlog*t*)_i_ can easily be transformed to the logarithmic ratio of extrapolated time limits *t*_0_ and *t*_m_, that is, *the stabilization period* log(*t*_m_/*t*_0_) defined by Kovacs in his earlier paper^4^
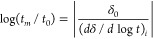
1where δ_0_ is the initial departure from equilibrium, immediately after a temperature jump from the temperature T_0_. The stabilization period describing the central part of the structural relaxation isotherm has been analyzed to some extent for one of the most frequently used phenomenological models involving nonexponentiality and nonlinearity.^5^

The stabilization period for the volume relaxation data shown in Figure 1 is about 2.5 times longer for down-jump than for up-jump. This *asymmetry of approach* is usually explained as a consequence of the nonexponentiality and nonlinearity contribution to the structure-dependent rate of relaxation during temperature down-jump and temperature up-jump experiments.^2^ Hutchinson^6^ pointed out that the volume contraction isotherms alone cannot distinguish between these contributions. The aim of this paper is to show that the nonexponentiality and nonlinearity contribution can be separated when the concept of the stabilization period is used. A detailed quantitative analysis of these two fundamental contributions to the asymmetry of approach to equilibrium for the most widely used phenomenological models of structural relaxation is provided, and a simple method for the estimation of nonexponentiality and nonlinearity parameters from the temperature down-jump and up-jump data is proposed.

Although this paper focuses mainly on the analysis of the volume relaxation data, the method of analysis based on the stabilization period is general and it can be applied to any type of isothermal structural relaxation data (enthalpy, index of refraction, density, dielectric loss or capacitance, etc.) involving symmetrical temperature jumps. The applicability of the proposed method, as well as the newly defined asymmetry ratio and isothermal relaxation rate, is thoroughly discussed for various glass-forming materials, ranging from organic polymers to inorganic glasses.

## 2 Phenomenological Models

There are three fundamental concepts that should be included in any successful model of the structural relaxation. Historically, the first concept was introduced by Tool^7,8^ who assumed that the relaxation time depends on both the temperature T and the actual structure is represented by the fictive temperature *T*_*f*_. It can be defined by an empirical equation:
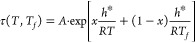
2where 0 < *x* ≤ 1 is the nonlinearity parameter, h*/R is the effective activation energy and *A* is an adjustable parameter. The prediction of the equilibrium state (*T*_*f*_ = *T*) by eq 2 provides an Arrhenius temperature dependence which does not agree with expected Vogel–Fulcher–Tammann (VFT) relation typical for viscous flow in supercooled liquids. An artificial partitioning of the actual and the fictive temperature contributions may also cause slightly distorted values of *A* and h*/R. It seems that entropy-based theories are more promising because they provide natural separation of *T* and *T*_*f*_. Based on the Adam–Gibbs^9^ cooperative relaxation theory, Scherer^10^ and Hodge^11^ derived the following expression for the relaxation time:
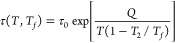
3where τ_0_ is a constant and Q = N_A_s* Δμ/k_B_C. The Adam–Gibbs quantities s* and Δμ are, the minimum entropy required for rearrangement and the activation energy for a single rearrangement, respectively. The parameter T_2_ is conceptually similar to the Fulcher or Kauzmann temperature and C is the extrapolated value of the configurational heat capacity at that temperature. In the equilibrium state above the glass transition where *T*_*f*_ = *T*, eq 3 yields the VFT equation with *T*_*0*_ = *T*_*2*_. Hodge^11^ has shown that eq 3 also provides relations between its parameters and the parameters of eq 2, i.e., T_2_/*T*_g_ ≅ (1 – x) and h*/R ≅ Q/x^2^.

The second essential concept was introduced by Narayanaswamy.^12^ According to his model, the structural relaxation should be linear in terms of the material (or reduced) time defined by
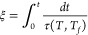
4

*Material time* quantifies how fast individual processes take place during physical aging, reflecting the existence of a material “inner clock.” Dyre^13^ used the nonlinear fluctuation–dissipation theorem to derive Narayanaswamy’s phenomenological theory of physical aging taking the material-time translational invariance as the basic assumption, providing answers to a number of interesting questions showing how the phenomenon of highly nonlinear aging can be reduced to a linear material-time convolution integral. Dyre^13^ suggested a possible pathway connecting this concept with material time in rheology. It is interesting to point out, that the concept of material time was also suggested by Hopkins^14^ for the stress relaxation description of viscoelastic substances under varying temperature (reported even earlier than the seminal papers of Kovacs).^1,4^ Recently, Douglas and Dyre^15^ proposed that the inherent harmonic mean-square displacement, emphasizing the role of the slowest particles, is probably the quantity that controls material time.

The third concept was introduced by Mazurin et al.^16^ and Moynihan et al.^17,18^ They combined Tool’s fictive temperature with the material-time concept and used the stretched exponential function, Φ, to account for the nonexponentiality of the structural relaxation
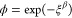
5where 0 < ß < 1 is the nonexponentiality parameter, inversely proportional to the width of a distribution of relaxation times. Equations 2, 4 and 5 represent the Tool–Narayanaswamy–Moynihan (TNM) formalism.^17,18^ In the Kovacs–Aklonis–Hutchinson–Ramos (KAHR) formalism,^19−21^ a discrete distribution of relaxation times is used, instead of eq 5. The expression for the relaxation time for the KAHR model is conceptually similar to eq 2. The Adam–Gibbs–Scherer–Hodge (AGSH) formalism^10,11^ uses eq 3 for the relaxation time partition between temperature and fictive temperature.

There is a continuing discussion of whether the relaxation kinetics based on the same fictive temperature can be applied to different physical properties. DeBolt et al.^18^ found that glassy B_2_O_3_ exhibits different relaxation kinetics for refractive index and enthalpy. Hodge^22^ in his review paper provided more examples and pointed out that “the temptation to equate T_f_ with a definite molecular structure should be avoided”. However, later it has been shown that for some inorganic glasses and organic polymers, the enthalpy and volume relaxation kinetics were experimentally indistinguishable within the glass transition region.^23^ For these systems it means that the fictive temperature change for volume and enthalpy seems to be similar within the experimental time scale.

## 3 Simulation of Structural Relaxation

The structural relaxation is usually simulated in terms of evolution of the fictive temperature for a well-defined time–temperature protocol capturing the entire thermal history of a glassy material from the very first moment of its departure from metastable equilibrium well above the glass transition. The structural relaxation can be described by combining the material-time concept (eq 4), and the Boltzmann superposition principle for any time–temperature protocol.^18,22^ For practical reasons, the protocol is divided into several consecutive steps involving either heating/cooling ramp or isothermal annealing. For the TNM model, the evolution of the fictive temperature during continuous heating or cooling at a rate q_k_ = d*T*/d*t* (negative for cooling) then can be expressed^18^ by eqs 6a and 6b:
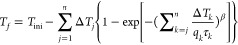
6a
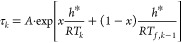
6bwhere T_ini_ is the initial temperature corresponding to the metastable equilibrium state, i.e., T_f_(T_ini_)= T_ini_. Equation 6a represents an implicit function as the expression for the relaxation time τ_k_ contains the variable recognized as the value of the function, that is, the fictive temperature at the end of the previous substep, T_f,k–1_.

The heating or cooling experiment is represented by small discrete temperature substeps Δ*T*_*k*_ ≈ 100 mK, calculating the fictive temperature at the end of each temperature step (eqs 6a, 6b). During isothermal annealing, the upper index of the Boltzmann summation is fixed and the material time summation argument Δ*T*_k_/q_k_τ_k_ is replaced by Δt_k_/τ_k_, where Δ*t*_*k*_ is a logarithmically spaced subinterval of the total annealing time. To ensure linearity, this subinterval must be small enough that T_f_ decays by less than about 100 mK between substeps. The isothermal evolution of the relative volume change δ (log *t*) after the temperature down-jump or up-jump, as shown in Figure 1 can be substituted for the calculated dependence of T_f_(log *t*). However, it is essential to emphasize that such calculation involves several consecutive steps, involving heating, cooling, and isothermal annealing, ensuring that a reliable data are obtained. As the time–temperature protocol and the calculation procedure are quite complex and resemble a real experiment, we use the term “computer simulated experiment” in this paper.

**Figure 2:**
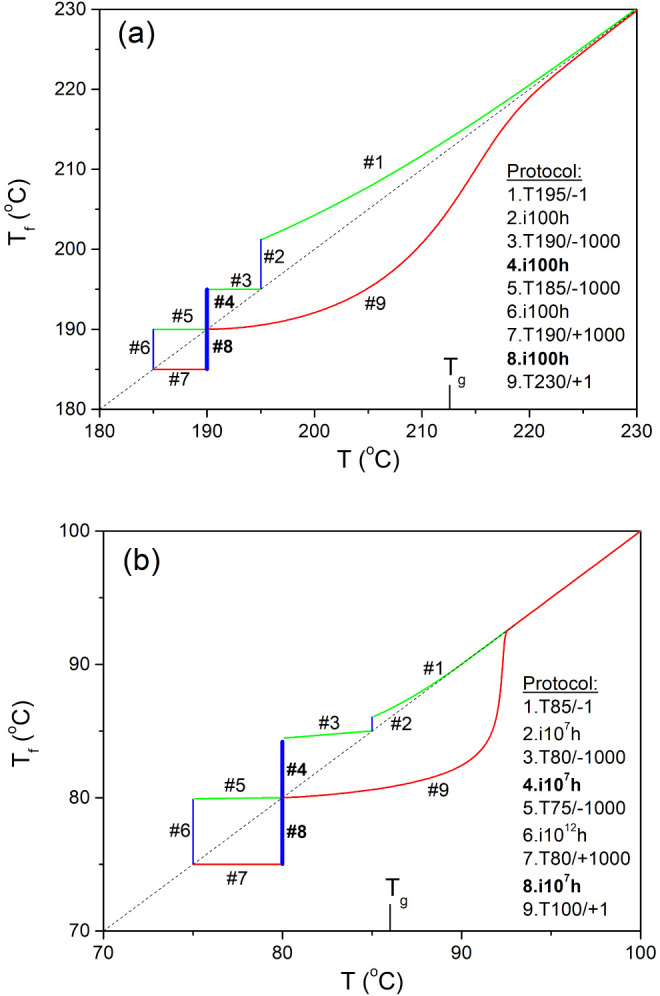
Evolution of the fictive temperature during the computer simulation experiment involving cooling in K/min (green lines), isothermal annealing in hours (blue lines), and heating in K/min (red lines) for: (a) As_2_S_3_ glass, (b) PVC polymer. The steps corresponding to the isothermal annealing after the temperature down-jump (**#4**) and up-jump (**#8**) are highlighted. The dashed line corresponds to the extrapolated equilibrium (T_f_ = T). For details, see text.

Figure 2 shows the fictive temperature evolution calculated for two very different materials: As_2_S_3_ glass^24^ and polyvinyl chloride (PVC),^11^ subjected to the temperature down-jump and up-jump of the same magnitude Δ*T* = ±5 K. The protocols (steps: #1–#9) for these computer simulation experiments are also shown in Figure 2. The TNM parameters used for the calculation of the heating/cooling steps and the isothermal annealing steps (eqs 6a, 6b) are summarized in Table 1. At the initial temperature T_ini_ (230 °C for As_2_S_3_ glass and 100 °C for PVC), i.e., well above the glass transition temperature, “the material” is in a metastable equilibrium where T_f_ = T_ini_. Slow cooling is then applied (q_k_ = −1 K/min) to the upper temperature limit T_0_ < *T*_g_ (step #1). In the next step, the material is isothermally annealed at T_0_ for a sufficiently long period of time (100 h for As_2_S_3_ glass and 10^7^ hours for PVC) to achieve equilibrium, i.e., T_f_ = T_0_ (step #2). After this equilibration at the temperature T_0_, the temperature down-jump (cooling rate q_k_ = −1000 K/min, Δt_0_ = 0.3s) to the temperature T is made (step #3). During the next annealing (100 h for As_2_S_3_ glass and 10^7^ hours for PVC) we obtain isothermal relaxation data for the temperature down-jump (step #4). After the equilibration at the temperature T (190 °C for As_2_S_3_ glass and 80 °C for PVC) a fast cooling (q_k_ = −1000 K/min) is applied to the lower temperature limit T_0_ (step #5). In the next step, the material is isothermally annealed at this temperature for a sufficiently long period of time (100 h for As_2_S_3_ glass and 10^12^ hours for PVC) necessary to achieve equilibrium, i.e., T_f_ = T_0_ (step #6). After this equilibration, the temperature up-jump (heating rate q_k_ = +1000 K/min, Δt_0_ = 0.3s) to the temperature T (190 °C for As_2_S_3_ glass and 80 °C for PVC) is made (step #7). During the next annealing (100 h for As_2_S_3_ glass and 10^7^ hours for PVC) we obtain isothermal relaxation data for the temperature up-jump (step #8). Finally, a slow heating rate (q_k_ = +1 K/min) is applied to return to the initial temperature T_ini_ (step #9).

**Table 1:** TNM Parameter Reported for Volume^24,25^ and Enthalpy^11,27^ Structural Relaxation of Glass-Forming Materials^a^

material	h*/R (kK)	–ln(*A*/s)	*x*	ß	σ (K^–1^)	ref
As_2_S_3_	32.4	62.1	0.31	0.82	0.09	(24)
a-Se	42.8	133.0	0.42	0.58	0.26	(27)
PVAc	71.3	227.7	0.35	0.57	0.49	(25)
PVC	225.0	622.0	0.10	0.23	1.57	(11)

a The parameter σ was calculated by eq 9.

**Figure 3:**
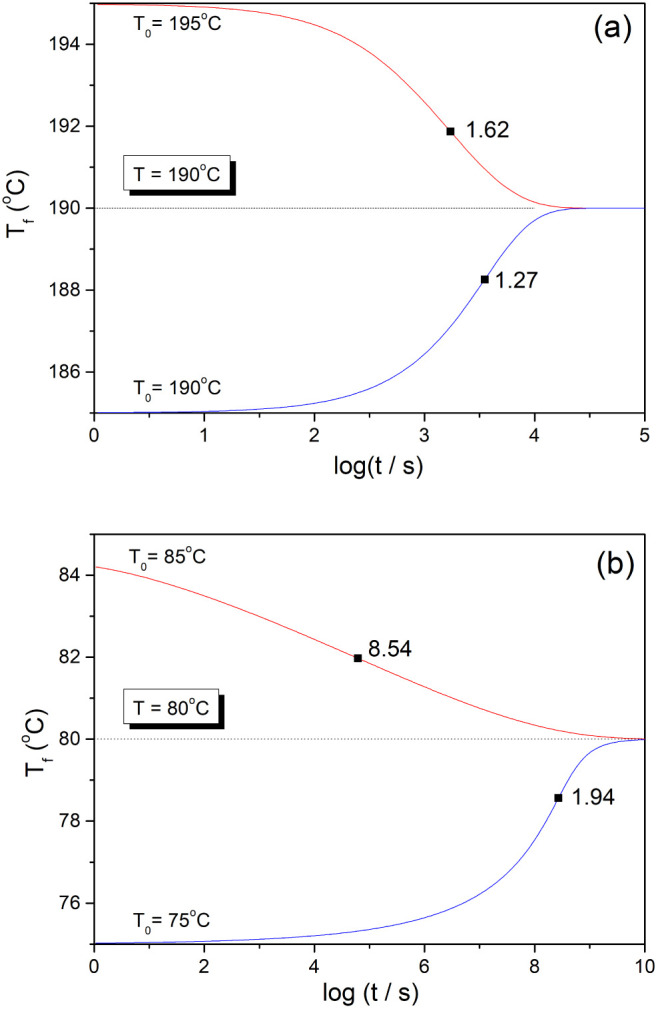
Asymmetry of the approach to equilibrium for: (a) As_2_S_3_ glass, (b) PVC polymer, subjected to computer simulated temperature down-jump (red solid lines) and up-jump (blue solid lines) of the same magnitude |Δ*T*| = 5 K. These lines were calculated using eq 6 (steps #4, #8, Figure 2) for the TNM parameters^24,11^ (Table 1). The stabilization period calculated as |Δ*T*/(d*T*_f_/d log *t*)_i_| is indicated by numbers next to the inflection points.

Figure 3 shows the asymmetry of the approach of the fictive temperature for As_2_S_3_ glass^24^ and polyvinyl chloride (PVC),^11^ subjected to computer simulated experiment for the temperature down-jump (Figure 2, step #4) and up-jump (Figure 2, step #8) of the same magnitude Δ*T* = ±5 K. The relaxation curves in this figure were calculated using eq 6 for the TNM parameters shown in Table 1 (see also Figure 2). The difference in the asymmetry of the approach is striking, though the magnitude of the temperature jump is identical. Similarly, the time scale to reach metastable equilibrium differs significantly, ranging from about 3 h for As_2_S_3_ glass to 300 years for PVC polymer.

There is another important aspect that should be mentioned. The fictive temperature immediately after the temperature jump T_f0_ should be close to *T*_0_. This is achieved for the computer simulated data for the As_2_S_3_ glass (Figures 2a, #3, #7 and 3a) or for the up-jump data for the PVC polymer (Figure 2b, #7). However, there is a difference of 0.79 K between these temperatures in the down-jump data for the PVC polymer (Figure 3b). This can be explained by the fact that the fictive temperature changes significantly during the temperature down-jump (Figure 2b, step #3), even that the thermal equilibration time is short (Δt_0_ = Δ*T*/q_k_ = 0.3s). Therefore, initial part of the aging curve is lost in this computer simulated experiment.

## 4 The Stabilization Period of Isothermal Relaxation

The simulated fictive temperature data as a function of the logarithmic time T_f_(log *t*) for As_2_S_3_ glass and PVC polymer shown in Figure 3 are similar to the experimental dilatometric data for PVAc polymer shown in Figure 1. This is not surprising as the relative volume change δ is proportional to fictive temperature. The stabilization period calculated as |Δ*T*/(d*T*_f_/d log *t*)_i_| for temperature down-jump differs substantially for these materials, ranging from 1.62 for As_2_S_3_ glass to 8.54 for PVC polymer. These values reflect significant differences in the TNM parameters for both materials (Table 1). From the mathematical condition for the inflection point of the relaxation function δ (log *t*) it was shown^5^ that the stabilization period after the temperature jump can be expressed as
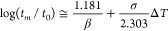
7where the numbers correspond to *e*/ln(10) and ln(10), respectively. The parameter σ quantifies the variation of the relaxation time on structural changes of the material at the inflection point of the isothermal relaxation curve and is defined as^23^
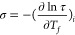
8

Taking the derivative of eq 2 according to eq 8, the parameter σ can be written for the TNM model as^23^
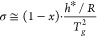
9and for the KAHR model as^23^
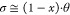
10where θ = h*/R*T*_g_^2^ is a reduced form of activation energy that ranges from about 0.1 K^–1^ for inorganic glasses to about 1 K^–1^ for polymers.^22^ Similarly, the parameter σ can be written for the AGSH model^23^
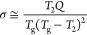
11

The parameter σ involves for TNM, KAHR or AGSH model the effective activation energy h*/R or its equivalent, the nonlinearity parameter (1 – x) or its equivalent and the glass transition temperature *T*_g_, that can be set for the relaxation time τ = 100 s in equilibrium (*T*_f_ = *T*).^22^ Then, for the AGSH model, eq 3 gives *T*_g_ ≅ *T*_2_ + Q/(ln 100 – ln τ_0_). Similarly, for the TNM model or the KAHR model, follows from eq 2: *T*_g_ ≅ (h*/R)/(ln 100 – ln *A*).

For the temperature down-jump the value of Δ*T* = T_0_ – T in eq 7 is positive (T_0_ > T). Therefore, the stabilization period [log(*t*_*m*_/*t*_*0*_)]_down_ is obtained by eq 7 as the sum of the nonexponentiality term (1.181/ß), and the nonlinearity term (σ/2.303) Δ*T.* In contrast, these two terms should be subtracted to obtain the stabilization period for the temperature up-jump [log(*t*_*m*_/*t*_*0*_)]_up_, as the value of Δ*T* in eq 7 is negative (T_0_ < T). It is remarkable that the nonexponentiality term, which represents the width of a relaxation time distribution, and the nonlinearity term representing the structural contribution to the relaxation time are clearly separated, even though they seem to be inextricably bound together by the material time.^6,22,23^ The first term in eq 7 is controlled just by the parameter ß. The second term is controlled by the parameter σ and it depends also on the magnitude of a temperature jump. Therefore, these two parameters that characterize the distribution of relaxation times ß, and their structure dependence σ, are key factors that determine the stabilization period of the isothermal structural relaxation. Their combined effect depends on the magnitude of the temperature jump.

For σ = 0 K^–1^ the stabilization period for down-jump and up-jump is identical. In such a case, the approach of both curves to equilibrium would be perfectly symmetric with the stabilization period equal to 1.181/ß. A very similar situation occurs for materials exhibiting very low σ value in combination with a temperature jump of a small magnitude. In these cases, the distribution of the relaxation times is again the main factor that determines isothermal structural relaxation. However, for σ ≈ 0.1–0.2 K^–1^ the structure dependence of the relaxation times becomes more important. Although, such low values are typical for inorganic glasses, significantly higher ones (0.5 < σ < 1.7 K^–1^) can be expected for some glass-forming polymers.^23^ The nonlinearity term (σ/ln 10)Δ*T* is equal to the nonexponentiality term *e*/(ß ln 10) if the following condition is fulfilled:
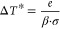
12where *e* is Euler’s number. The magnitude of the temperature up-jump |Δ*T*| should be lower than ΔT* to ensure a positive stabilization period, i.e., [log(*t*_m_/*t*_0_)]_up_ > 0. However, the maximum of |Δ*T*| for any temperature jump should be set at somewhat lower value, i.e., |Δ*T*| < 0.7ΔT*.

**Figure 4:**
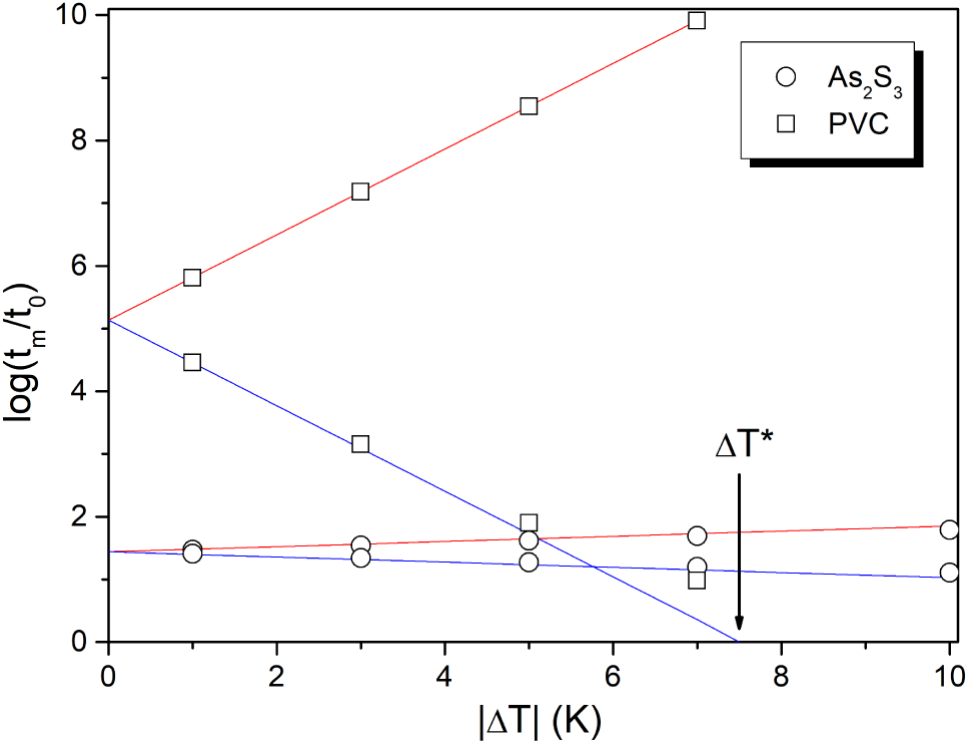
Stabilization period as a function of |Δ*T*| for As_2_S_3_ glass and PVC polymer. Lines were calculated for down-jump (red lines) and up-jumps (blue lines) using eq 7 for ß and σ parameters reported in Table 1. The points represent the stabilization period estimated as |Δ*T*/(dT_f_/d log *t*)_i_| from relaxation curves simulated by eq 6 for the same set of TNM parameters (see Section 3). Arrow indicate ΔT* for PVC, calculated by eq 12.

Figure 4 shows the stabilization period for the structural relaxation of As_2_S_3_ glass and PVC polymer as a function of Δ*T* predicted by eq 7 (full lines) for *ß* and σ parameters shown in Table 1. The points represent the stabilization period estimated as log(*t*_*m*_/*t*_*0*_) = |Δ*T*/(d*T*_f_/d log *t*)_i_| for data shown in Figure 2 (|Δ*T*| = 5 K) and similar relaxation curves calculated by eq 6 for different |Δ*T*| (see Section 3). These estimated values agree well with the prediction of eq 7, except for the up-jump for PVC polymer at Δ*T* = −7 K. In this case, the temperature jump is higher than the applicability limit of eq 7, i.e., |Δ*T*| < 0.7ΔT* (where ΔT*= 7.5 K for PVC polymer). No such limitation is observed for the As_2_S_3_ glass, as within the scale of Figure 3 the condition |Δ*T*| < 0.7ΔT* is fulfilled (where ΔT* = 36.8 K for the As_2_S_3_ glass). It is interesting to point out that the time scale of the stabilization period for PVC down-jump and up-jump spans 10 orders of magnitude just for a ± 7 K deviation from 80 °C, as evident from Figure 4.

## 5 Discussion

Although the structural relaxation is highly nonlinear, and its mathematical description is complex, involving the convolution integral that retains the whole thermal history from the very first departure from equilibrium, the equation for the stabilization period derived at the inflection point of the relaxation curve is quite simple and linear as described by eq 7 for temperature down-jump and up-jump. It has been shown earlier in the previous section that this equation can be used, when |Δ*T*| < 0.7ΔT*. It is convenient to define quantities: S^+^ = [log(*t*_m_/*t*_0_)]_down_+[log(*t*_m_/*t*_0_)]_up_ and S^–^ = [log(*t*_m_/*t*_0_)]_down_ – [log(*t*_m_/*t*_0_)]_up_ for the temperature down-jumps and up-jumps of the same magnitude |Δ*T*|. The nonexponentiality parameter ß and the parameter σ representing the structural change of the relaxation time then can be estimated by following equations:
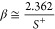
13a
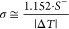
13bwhere the numbers correspond to 2*e*/ln(10) and ln(10)/2, respectively. If these equations are applied to the stabilization period calculated as |Δ*T*/(dT_f_/d log*t*)_i_| for the computer simulated relaxation data shown in Figure 3 we get the following parameter values: ß = 0.82 and σ = 0.08 K^–1^ for As_2_S_3_ glass, and ß = 0.23 and σ = 1.52 K^–1^ for PVC polymer. These values agree very well with the TNM parameter sets used for the computer simulations (see Table 1).

Similarly, we can estimate by eq 13 the following parameter values for the stabilization period for Kovacs^1^ PVAc data for the temperature down-jump and up-jump (|Δ*T*| = 5 K) shown in Figure 1: ß = 0.57 and σ = 0.42 K^–1^. Sasabe and Moynihan^25^ reported the enthalpy relaxation data of PVAc. These authors determined the TNM parameters by fitting the heat capacity data curve for nonisothermal differential scanning calorimetry (DSC) experiments, providing: ß = 0.57 and σ = 0.49 K^–1^. Hodge^22^ estimated that this fitting procedure involves uncertainties in ß and *x* of about ±0.05. The parameter *ß* for PVAc is identical and the parameter σ is within these combined limits. Nevertheless, it should be taken into account that Sasabe and Moynihan^25^ and Kovacs^1^ used different samples of PVAc polymer for their experiments. The thermal history and the time–temperature protocol were also different. Although the initial temperature is not as important for a complex DSC experiment involving one or multiple thermal cycles, its right selection is essential for any dilatometric temperature jump experiment. Figure 1 clearly shows that the initial departure from equilibrium for the temperature down-jump |δ_0_|= 1.37 is significantly lower than that for the temperature up-jump |δ_0_|= 2.30. To explain this difference (about 40%), it is convenient to simulate the Kovacs temperature jump experiment for the TNM parameters obtained by Sasabe and Moynihan.^25^

**Figure 5:**
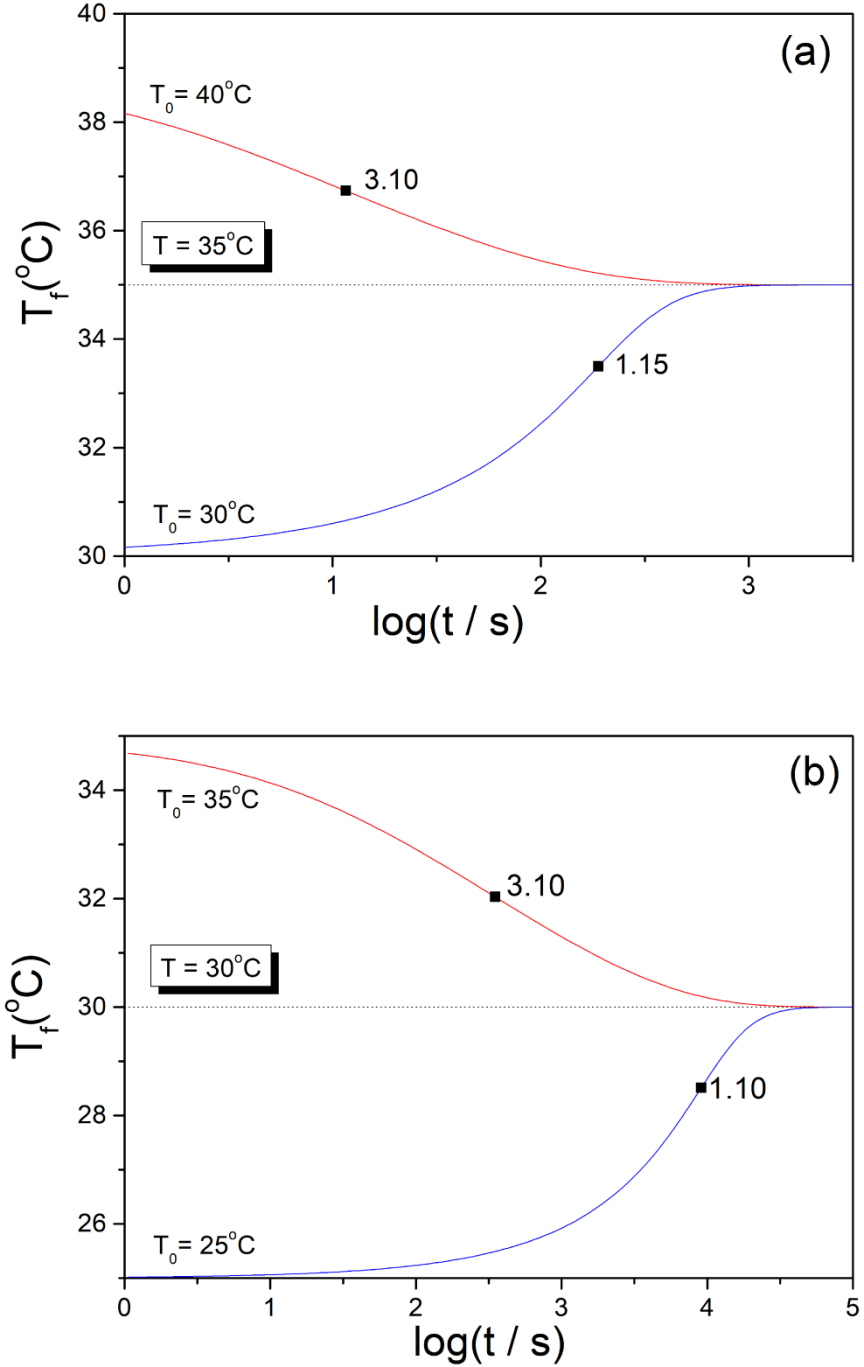
Asymmetry of the approach to equilibrium for PVAc polymer subjected to computer simulated temperature down-jump (red solid lines) and up-jump (blue solid lines) of the same magnitude |Δ*T*| = 5 K and (a) *T* = 35 °C, (b) *T* = 30 °C. These lines were calculated using eq 6 as described in Section 3 for the TNM parameters^25^ (Table 1). The stabilization period calculated as |Δ*T*/(d*T*_f_/d log*t*)_i_| is indicated by numbers next to the inflection points.

Such computer-simulated data using a similar procedure as described in the Section 3 are shown in Figure 5a. It is evident that the simulated relaxation response to the temperature down-jump and up-jump for the TNM parameters obtained from the DSC data (Figure 5) is very similar to the dilatometric data reported by Kovacs^1^ (Figure 1). The fictive temperature corresponding to the initial departure from equilibrium for the temperature down-jump *T*_f_ = 38.16 °C is significantly lower than it should be (*T*_f0_ = *T*_0_ = 40 °C). This fictive temperature drop represents about 37% of Δ*T* and it is clearly caused by the structural relaxation taking place during thermal equilibration associated with the temperature down-jump from *T*_0_ (40 °C) to *T* (35 °C).

We have shown earlier that for our computer simulation we can expect that the thermal equilibration time due to the temperature jump is Δ*t*_0_ = Δ*T*/q_k_ = 0.3 s. It is impressive that more than one-third of the relaxation data is lost during such a short time interval. Kovacs^1^ reported a significantly longer thermal equilibration time for his mercury dilatometric experiment (Δ*t*_0_ ≅ 36 s). Therefore, we can assume that the 40% drop in his PVAc dilatometric data is probably due to loss of relaxation data during thermal equilibration of the dilatometer. We can avoid these problems by shifting *T*_0_ and *T* to lower values.

Figure 5b shows the simulated data for the temperature jump experiment of the same magnitude at temperature *T* = 30 °C. As expected, a nearly complete relaxation curve is obtained for the temperature down jump and equilibrium is attained after a longer time. Using the stabilization period shown in Figure 5b, eq 13 provides values of ß = 0.56 and σ = 0.46K^–1^ that are close to those reported by Sasabe and Moynihan.^25^ The selected magnitude of the temperature jump |Δ*T*| = 5 K is close to 0.7ΔT* (where ΔT* = 9.7 K for PVAc polymer) which explains larger error in the estimation of the parameters. It would be interesting to test some unpublished Kovacs data^26^ for similar initial and final temperature.

**Figure 6:**
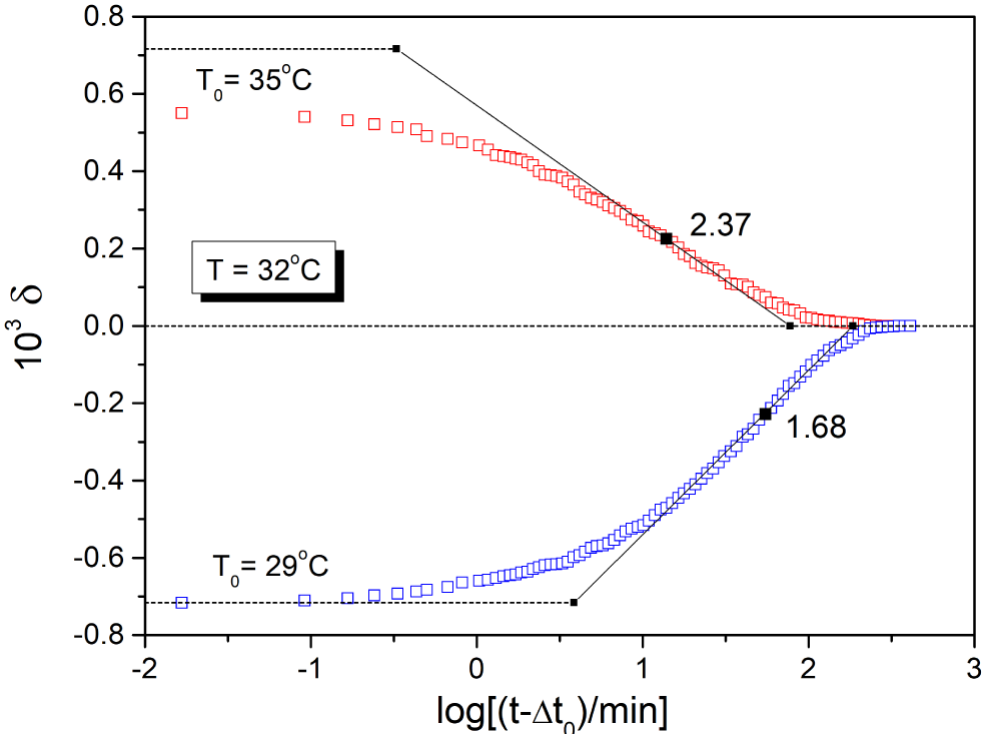
Asymmetry of the approach to equilibrium for amorphous selenium (a-Se) subjected to a temperature down-jump and up-jump of the same magnitude |Δ*T*| = 3 K. Data (points) were taken from ref. 27, Δ*t*_0_ = 70s is the thermal equilibration time. The dotted lines indicate the initial departure δ_0_ at *T*_0_ as well as the equilibrium line at temperature *T* = 32 °C. Solid lines correspond to the inflectional tangent of the relaxation curve. The numbers shown next to the curves correspond to the value of the stabilization period calculated by eq 1.

Figure 6 shows dilatometric data for amorphous selenium^27^ (|Δ*T*| = 3 K) obtained by a similar experiment to the one used for the first time by Kovacs.^1^ The initial temperature for the down-jump (*T*_0_ = 35 °C) is reasonably selected. However, even in this case the initial departure from equilibrium for the temperature down-jump |δ_0_| = 0.56 is lower than that for the temperature up-jump |δ_0_| = 0.71. This difference (about 20%) is probably due to loss of relaxation data during the thermal equilibration time of the dilatometer (Δ*t*_0_ ≅ 70 s).^27^ The magnitude of the temperature jump is much lower than 0.7ΔT* (where ΔT* = 18.0 K for a-Se). Therefore, we can expect that the stabilization period provides a solid basis for the estimation of both parameters. Equation 13a and 13b gives the following values: ß = 0.58 and σ = 0.26 K^–1^ for a-Se. These values are identical to the TNM reported earlier^27,28^ for the volume relaxation of this material (see also Table 1). It seems that careful selection of the initial temperature (*T*_0_ < *T*_g_) as well as achievement of a truly equilibrium state at this temperature is essential for a reliable temperature jump experiment.

It is convenient to define the asymmetry ratio, quantifying the approach to equilibrium after the temperature down-jump and the temperature up-jump of the same magnitude |Δ*T*| as A_R_ = [log(*t*_m_/*t*_0_)]_down_/[log(*t*_m_/*t*_0_)]_up_. Using eq 7, it can be expressed as
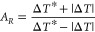
14where ΔT* is defined by eq 12. Equation 14 allows to estimate the following A_R_ values for all amorphous materials discussed here: 1.3 for As_2_S_3_ glass, 1.4 for a-Se, 3.1 for PVAc, and 4.9 for PVC. The asymmetry ratio of the stabilization period calculated from experimental or computer-simulated data for the temperature down-jump and up-jump gives 1.3 (Figure 3a), 1.4 (Figure 6), 2.5 (Figure 1), and 4.4 (Figure 3). These values are in reasonable agreement with the estimated ones (eq 14), taking into account uncertainties related to the selection of T_0_ (for PVAc) or proximity |Δ*T*| ≈ 0.7ΔT* (for PVC) discussed earlier.

**Figure 7:**
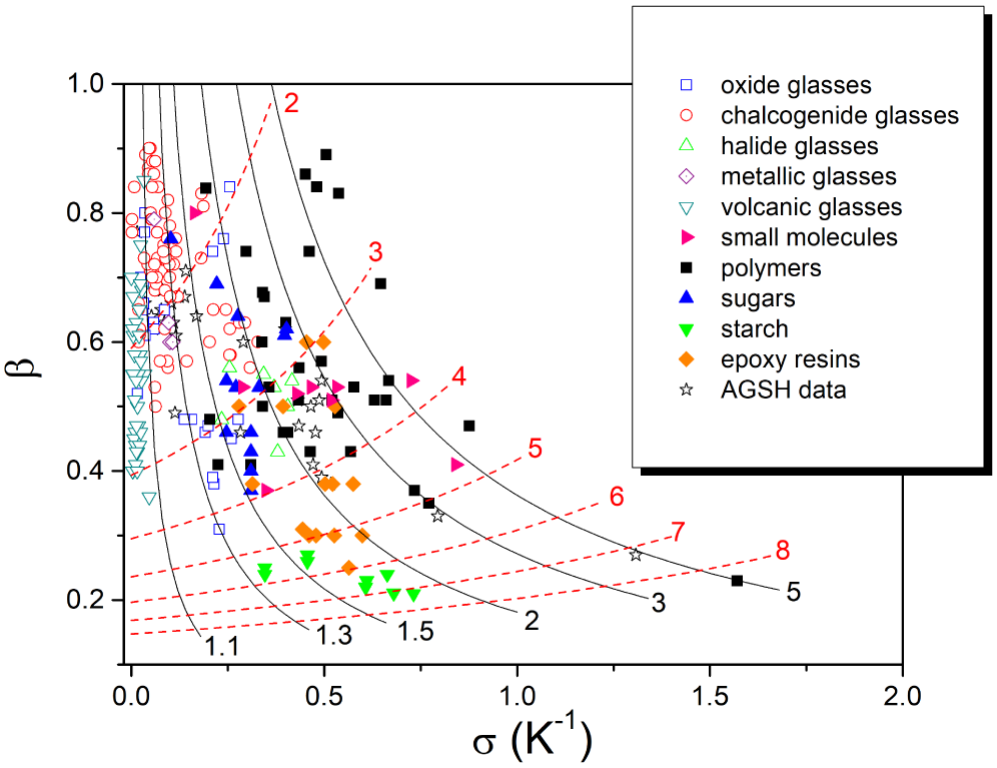
Value of *ß* and σ parameters for reported TNM (KAHR) data for oxide glasses,^10,18,23,29−32^ chalcogenide glasses,^33^ halide glasses,^22^ metallic glasses,^34,35^ volcanic glasses,^36,37^ organic polymers,^11,22,27,38−46^ sugars,^47−50^ starch,^51,52^ epoxy resins,^53−60^ and small molecules,^61−64^ as well as AGSH data^10,11,25,51,65−67^ for various amorphous materials. Red dashed lines indicate the stabilization period [log(*t*_*m*_/*t*_*0*_)]_down_, calculated by eq 7 for Δ*T* = 5 K. The solid black lines correspond to the fixed value of the asymmetry ratio A_R_, calculated by eq 14 for |Δ*T*|= 5 K. The numbers next to the curves indicate [log(*t*_*m*_/*t*_*0*_)]_down_ (red) and A_R_ (black).

The TNM model has been applied for the description of physical aging in many different glass-forming materials. All these materials were mostly studied by nonisothermal enthalpy aging experiments involving temperature ramps or more complex thermal history. They are characterized by four parameter sets (ß, h*/R, ln *A*, and *x*), which describe the nonisothermal structural relaxation. However, we have shown earlier that for the description of isothermal relaxation data by the stabilization period, only two parameters characterizing the distribution of relaxation times *ß*, and their structure dependence σ are needed. The parameter σ, in fact, combines h*/R, *x* and ln *A* (through *T*_g_) for the TNM model and equivalent parameters for the KAHR and AGSH model as defined by eqs 9–11. The points in Figure 7 show ß and σ data obtained in this way for a collection of more than 270 sets of TNM (or KAHR) parameters reported for glass-forming materials ranging from inorganic systems such as oxide glasses,^10,18,23,29−32^ chalcogenide glasses,^33^ halide glasses,^22^ metallic glasses,^34,35^ and natural volcanic glasses^36,37^ to polymers,^11,22,27,38−46^ sugars,^47−50^ starch,^51,52^ epoxy resins,^53−60^ and small organic molecules.^61−64^ The data obtained from the AGSH parameters^10,11,25,51,65−67^ characterizing some glass-forming materials are also included.

The dashed lines shown in Figure 7 indicate the stabilization period for the structural relaxation response after the temperature down-jump (Δ*T* = 5 K) calculated by eq 7. An important part of oxide, chalcogenide, metallic, and volcanic glasses exhibit fast relaxation^68^ with the stabilization period within two or three decades of time. These inorganic systems are usually characterized by relatively low fragility indicating the sensitivity of the structure to a temperature change, or in Angell^69^ words: “··· which, with little provocation from thermal excitation, reorganize to structures that fluctuate over a wide variety of different particle orientations and coordination states”. In contrast, polymers with longer entangled chains, some epoxy resins, and starch-water systems exhibit rather slow relaxation,^68^ with the stabilization period longer than five decades of time. These systems seem to have built-in resistance to structural changes that prolong their relaxation response to the temperature jumps.^69^

The full lines in Figure 7 indicate the constant asymmetry ratio A_R_ quantifying the asymmetry of the approach to equilibrium calculated by eq 14 (for |Δ*T*| = 5 K) in the range 1.1 ≤ A_R_ ≤ 5. It is seen that the approach to equilibrium after the temperature down-jump and up-jump is nearly symmetric (A_R_ < 1.3) for natural volcanic glasses, metallic glasses, and for an important part of chalcogenide glasses. On the other hand, we can expect a clearly asymmetric approach to equilibrium (A_R_ > 2) for organic molecular systems, epoxy resins, and polymers. For some systems such as polyethylene terephtalate,^40^ polystyrene,^42,46^ and *o*-terphenyl^61^ selected magnitude of the temperature jump (|Δ*T*| = 5 K) seems to be too high, and the condition |Δ*T*| *<* 0.7ΔT* might not be fulfilled.

The experimental data shown in Figure 7 illuminate the differences in the kinetics of structural relaxation among chemically dissimilar systems, clearly showing the effect of the parameter characterizing the distribution of the relaxation times (*ß*) and of the parameter characterizing the structural dependence of the relaxation time σ = −(∂lnτ/∂*T*_f_)_i_. However, we should be aware that the experimental data included in this figure were obtained under different experimental conditions. In particular, the thermal history and peculiarities of experimental setup such as time constants and precision of temperature control might be important. The promising experimental setup for nearly ideal temperature jump experiments (Δ*t*_0_ ≅ 2 s, temperature fluctuations below 100 μK) has been described by Hecksher et al.^70^ and Riechers et al.,^71^ allowing one to precisely monitor the imaginary part of dielectric loss or capacitance at a fixed frequency. These techniques probably would be suitable for more extensive testing applicability limits of eq 7, as well as the proposed method of estimation ß, σ and the asymmetry ratio from temperature down-jump and up-jump experiments at identical Δ*T*.

The material time is an essential concept for structural relaxation in any glass-forming material. It may be thought of as the time measured on a clock whose rate changes during the relaxation process. Although, the material time ξ can be calculated (eq 4) from experimental data such as the refractive index,^12^ density,^10^ sample length,^24,72^ complex heat capacity^73^ and complex capacitance,^74^ its experimental determination has not been realized for a long time. Nevertheless, Böhmer et al.^75^ recently demonstrated a direct measurement of the material time after a temperature jump experiment of a molecular glass using the time-autocorrelation function of the intensity fluctuations probed by multispeckle dynamic light scattering. According to Narayanaswamy,^12^ the material time determined using the time evolution of a material property during the temperature jump experiment should collapse to a single relaxation curve constructed as a normalized function of ξ for any magnitude of the temperature jump. The stabilization period of this normalized relaxation curve is then expressed as log(ξ_m_/ξ_0_) ≅ 1.181/ß.

## 6 Conclusions

We have shown that the temperature down-jump and up-jump experiments of the same magnitude |Δ*T*| to the same temperature provide a suitable basis for the separation of nonexponentiality and nonlinearity of the structural relaxation. These experiments can be quantitatively described using the stabilization period on the logarithmic time scale, which is defined as the sum of the nonexponentiality term 1.181/*ß* and the nonlinearity term (σ/2.303)Δ*T* for the temperature down-jump, and as their difference for the temperature up-jump. The characteristic material parameter σ = −(∂lnτ/∂*T*_f_)_i_ quantifies the variation of the relaxation time on structural changes at the inflection point of the relaxation curve and is formulated for the TNM, KAHR and AGSH phenomenological model of structural relaxation. Although physical aging is highly nonlinear, and its mathematical description is complex, involving the material time concept, which retains the entire thermal history of the material under study, the equation for the stabilization period is quite simple and linear.

A convenient method for estimation of the ß and σ parameters from experimental data is proposed. The applicability of this approach is tested using previously reported experimental data, as well as by calculated isothermal data for aging of polymers and other glass-forming materials. This concept helps to understand structural relaxation kinetics in a simple and consistent manner.
